# Predictors of skilled assistance seeking behavior to pregnancy complications among women at southwest Ethiopia: a cross-sectional community based study

**DOI:** 10.1186/s12978-015-0102-z

**Published:** 2015-11-28

**Authors:** Serawit Lakew, Erdaw Tachbele, Terefe Gelibo

**Affiliations:** Department of Nursing and Midwifery, Arba Minch College of Health Sciences, Arba Minch, South West Ethiopia; Department of Nursing and Midwifery, Addis Ababa University, Addis Ababa, Ethiopia; Department of Public Health, Addis Ababa Science and Technology University, Addis Ababa, Ethiopia

**Keywords:** Predictors, Skilled assistance, Pregnancy complications, Women, South west Ethiopia

## Abstract

**Background:**

In Ethiopia, about 20,000 women die each year from complications of pregnancy and child birth with many more maternal morbidities occurring for each maternal deaths. This makes Ethiopia one of the highest countries for maternal deaths in the developing world. This study attempted to assess women’s skilled assistance seeking behaviour for pregnancy complications among those who gave birth.

**Method:**

A cross-sectional community based study was conducted among women who gave birth within one year regardless of their delivery place. The study was carried out in fifteen randomly selected villages at Arba Minch Zuria district, south west Ethiopia. Data was collected house-to-house using a pretested Amharic questionnaire. During the survey, 798 women were interviewed. Logistic regression model was applied to control confounders.

**Results:**

Out of the total sample, 344 (43.1 %) respondents reported at least any one of the pregnancy complications faced in the recent pregnancy. The most common complications reported were malaria (57 %), nausea/vomiting (47.1 %) and severe head ache (29.1 %). of those women who faced complications, around 254 (73.8 %) sought assistance from a skilled provider. Ninety (26.2 %) of the respondents sought assistance either from unskilled provider or home based self-care. Unable to understand the seriousness of the complications, thought as unnecessary, and family disapproval were the major reasons for not seeking care from skilled providers. Belonging to monthly household income $US25- 100 (AOR = 3.4, 95 % CI; 1.04, 11.4), getting antenatal care from a skilled provider (AOR = 10.6, 95 % CI; 3.3, 34.5), Women in the age 20–34 years old (AOR = 3.8; 95 % CI, 1.2, 12.3), Availability of transport access (AOR = 72.2; 95 % CI; 17.2, 303.5) were significantly associated with seeking assistance from a skilled provider.

**Conclusions:**

Nearly half (43.1 %) of the women had faced pregnancy complications to the recent birth of last one year. Majority (2/3^rd^) of the women who reported complications sought skilled assistance. Family, income, transport issue and antenatal care use were independent predictors for skilled assistance from skilled provider.

## Background

An estimated 287,000 maternal deaths occurred in 2010 worldwide, which is 50 % reduction from 1990 baseline [1]. In Ethiopia, about 20,000 women die each year from complications of pregnancy and child birth with many more maternal morbidities occurring for each maternal death. This makes Ethiopia to be one of the countries with the highest maternal mortality ratio, which is 676 per 100,000 live births, EDHS 2011 Report [2].

All pregnant women are at risk of developing life-threatening complications and around 15 % of these women develop complications [3]. These complications can involve the mother’s health, the baby’s, or both. Pregnancy complications can range from mild and annoying discomforts to severe, sometimes life-threatening, illnesses [4]. Some of these life threatening complications range from vaginal bleeding up to high fever. And non- life threatening complications range from nausea and/or vomiting to varicus vein [4–8].

The outcomes of most pregnancy complications are unpredictable unless they are managed by appropriate health care providers. Studies indicated that life threatening maternal complications may end up in the death of the mother within two hours of onset in postpartum hemorrhage to six days in puerperal sepsis [9]. Previous studies have identified factors predicting the care seeking behaviors for pregnancy complications include higher educational status, ANC utilization, near distance to health facility, availability of transport system, small family size, previous pregnancy experience, and good economic status have been observed with high probability of skilled assistance seeking for pregnancy complications [2, 5, 6, 9–16]. According to the latest Ethiopian demographic and health survey report, skilled maternal health services utilization is still low. So, this study was aimed to determine skilled assistance seeking behaviour for pregnancy complications that women faced (for any one or more complications) among those who were permanent dwellers of Arba Minch Zuria district, Gamo Gofa Zone.

## Methods

### Study setting and design

A community based cross-sectional study was conducted from April 15 to Jun 5, 2014 in Arba Minch Zuria district, Gamo Gofa Zone, Southwest Ethiopia, which is found in the Great Rift Valley, located 505 kms south of Addis Ababa [17]. In the district there were 30 kebeles and a total population of 195,315, of whom 95,704 were men and 99,611 women [18]. The district had 38,663 total estimated households and 9,766 estimated population of women with under one year child or still birth in the last one year prior to this survey (2.4 % for <1 year and 2.6 % for still births, in the total population). The health infrastructure of the district includes six health centers, thirty health posts, one Hospital of the governmental, and fifteen medium level and lower level private clinics [17–19].

### Sample size and sampling procedure

The sample size was determined taking 52.1 % estimated prevalence of skilled assistance at Northwest Ethiopia (since northwest had nearly similar health infrastructure, socio-economy and culture with southwest) [9]. Assuming 5 % margin of error, 95 % confidence level, 5 % non-response rate, and design effect, the total sample size was 804. For sampling, multistage cluster sampling technique was used. Out of 30 clusters of kebeles (kebele is the lowest administrative unit in Ethiopia) in the district, 15 kebeles were randomly selected for this study (that is 50 % of the cluster, clusters were assumed homogenous to the study variables). Within each of these 15 kebeles, 3 villages (Villages are sub-divisions of one kebele) were selected. This is, again, 50 % selection out of an average six locations (villages). Sampling frame of household was obtained for each kebeles from district administration Health Office. For selection of respondents in each kebeles and villages, Population proportionate to size (PPS) technique was employed. The respondents from the sampling frame of the eligible households were selected by lottery method. One center of the village was selected in each village. From this center spinning a pen technique was used for selection of walking direction by data collectors. The household in the direction of Tip of pen was started with a nearby first. After a successful interview of each household, the interviewer continued to the immediate nearby household of the eligible and selected woman until the required sample size was achieved in each of the kebele.

### Measurement

Data were collected using interviewer administered questionnaires. The data collection tool was adapted from DHS and other literatures on maternal health surveys [6]. The main foci of the questionnaire were on socio-demographic characteristics; knowledge and experiences related to pregnancy complications; and use of skilled maternal care services. Some of questions presented include: the type of facility visited, type of care provider; decision maker to seek care; transport access; and distance from home to health facility. A maximum effort was made to ensure privacy during interview. Data collectors and supervisors were hired from nursing background. They have given five days training on data collection techniques and objectives of the study. The interviews were made in Amharic local language. Translations and re-translations were performed by experts of both English and Amharic language.

### Statistical analysis

The data were coded, edited and entered into Epi-info version 3.5.2, cleaned and analyzed by SPSS for windows version 20. Frequencies, proportions and summary statistics were used to describe the study population in relationship to the relevant variables. Statistical analysis had three steps: first association was done between potential predictors of socio-demography and Skilled Antenatal visit (by Doctor, Nurse, or Midwife) for skilled assistance seeking to pregnancy complications using bivariate analysis and 95 % confidence intervals to show existence of bivariate association. Next, to identify the independent contribution of each variable multivariate logistic regression model was used for the variables having association (*p* < 0.05) in bivariate logistic regression model. Finally, it was evaluated that variables identified as associated (*P* < 0.05) with the outcome variable in the multivariate analysis were used to predict the existence of association.

### Data quality control

Data Collection tool was adjusted for locally suitability and pretested. Every day completed questionnaires were reviewed and checked for completeness and relevance by the supervisors and Principal investigators. All the necessary feedback was offered to data collectors in the next morning before the actual procedure. Data checked for completeness, coded, entered into computer, cleaned and frequency checked for outliers and missing values before analysis.

### Ethical issues

Ethical clearance was obtained from Addis Ababa University, School of Allied Health Sciences Institutional Review Board (IRB). The study was commenced after letter of cooperation written to kebele Health Extension Workers (HEW) from District Health Office. Informed verbal consent was secured for each study subjects. Each respondent was informed about the objective of the study and assurance of confidentiality, risks and benefits.

### Definitions

**Income** = measured based on respondents estimated household monthly income.

**Transport access** = includes availability of roads and vehicle transport (bajaj, ambulance, car, or bus), but mule transport excluded.

**Knowledge of complications** = respondents who responded the median and more of the complications from the list for knowledge were designated as knowledgeable and those below median were not knowledgeable.

**Sought skilled assistance** = respondents who sought assistance from Doctors, Nurses, Midwifes, or Health Officers for any one of the recent pregnancy complications.

## Results

### Socio-demographic characteristics of the study participants

A total of 798 women participated in the survey making the response rate of 99 %. Majority (78.2 %) of the women at the time of last pregnancy were 20–34 years of age with mean age of 25.7 years ± 6.3 standard deviation and range of 14–49 years of age. The dominant ethnicity in the surveyed area was Gamo (73.4 %), followed by Ganta (13 %) and Welayta (11.5 %) ethnic groups respectively. Majority (99.7 %) of the respondents were a Christian religion follower that belongs to either Christian orthodox (28.3 %) or Christian protestant (71.4 %). The median monthly income of the family was $US45 (Table 1).

**Table 1:** Frequency and percentage distribution of respondents according to selected socio-demographic characteristics, Arba Minch Zuria district, south west Ethiopia, 2014

Characteristics		Frequency (n), *n* = 798	Percentage (%)
Age (years)	15–19	96	12.0
	20–34	624	78.2
	35–49	78	9.8
	Mean ± std.dev^a^	25.7 ± (6.3)	
Religion	Protestant	570	71.4
	Orthodox	226	28.3
	Others^b^	2	0.3
Ethnicity	Gamo	586	73.4
	Ganta	104	13.0
	Welayta	92	11.5
	Others^c^	16	2.0
Marital Status	Married	770	96.5
	Others^d^	28	3.5
Family Monthly Income ($US)	<25	300	37.6
	25–100	394	49.4
	>100	104	13
	Median	45	
Women Education	No Education	192	24.1
	Primary	442	55.4
	Secondary	124	15.5
	College/University	40	5
Birth Order	1	230	28.8
	2–3	394	49.4
	4–5	126	15.8
	6+	48	6
	Median	2	
No transport access problem^e^	Yes	202	62.7
	No	120	37.3
	Missing^f^	476	59.6

N.B: ^a^standard deviation, ^b^muslim, ^c^Zayse, Oromo, Amhara and Ganjule, ^d^widowed, divorced, single, ^e^transport access is for motor vehicle transport, existence of road to the facility, (foot and mule transport excluded), ^f^respondent who did not face complications were not asked for access, transport access is difficult to say yes or no(not adequate, but sometimes used) were also under missed

### Complications women faced

**Fig. 1:**
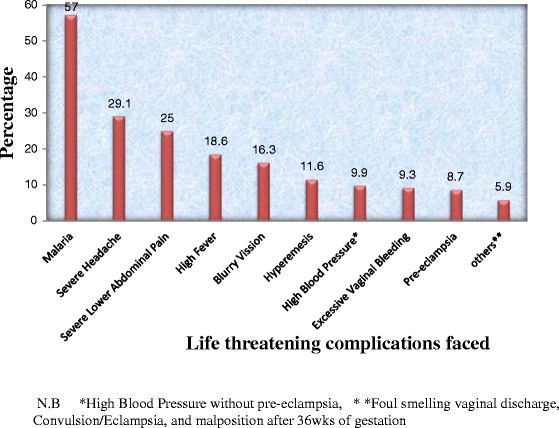
Percent distribution of life threatening pregnancy complications reported in the recent pregnancy, Arba Minch Zuria District, South West Ethiopia, 2014

**Fig. 2:**
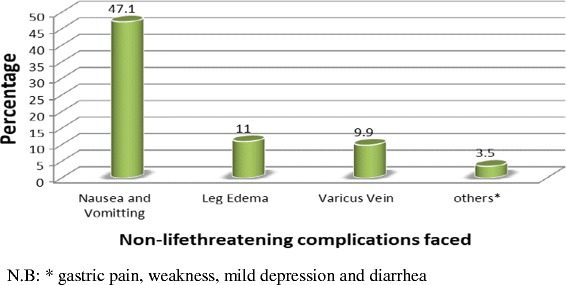
Percentage distribution of non-life threatening pregnancy complications reported in the recent pregnancy, Arba Minch Zuria District, South West Ethiopia, 2014

Out of 798 respondents included in the study, 43.1 % (344 out of 798 women) had encountered at least any one of the problems either life threatening or non-life threatening or both, last during the recent pregnancy in the last 12 months. Of this complications, about 334 (97.1 %) reported life threatening pregnancy complications and 180 (52.3 %) non-life threatening pregnancy complications (since most respondents had mixed complications percentage was greater than 100 and number greater than 344). Mean (±SD) occurrence 2 ± 1 for life threatening pregnancy complications and mean (±SD) occurrence 0.72 ± 0.8 for non-life threatening pregnancy complications. The major life threatening complications faced at the last pregnancy were malaria 196 (57 %); severe headache 100 (29.1 %); and severe lower abdominal Pain 86 (25 %) (Fig. 1). Nausea and vomiting occurred on 162 (47.1 %) of mothers, which was the larger non-life threatening pregnancy complication (Fig. 2).

### Women assistance seeking behaviour

Out of women faced complications, 73.8 % (254 out of 344) sought skilled assistance, 16.9 % (58 out of 344 women) sought unskilled assistance (sought assistance from unskilled provider, such as HEW, TBA, and other unqualified health worker), and the remaining 9.3 % (32 out of 344 women) did seek no assistance at all or had home based self-care (Table 2).

**Table 2:** Percent distribution of the respondents by seeking health care assistance to the complication faced last in the recent pregnancy, Arba Minch Zuria district, south west Ethiopia, 2014

Characteristics		Seeking health care assistance			
		Skilled assistance^a^	Unskilled assistance^b^	No-assistance^c^ (self-care)	Total
		*n* = 254 (73.8 %)	*n* = 58 (16.9 %)	*N* = 32 (9.3 %)	*n* = 344 (100 %)
Age (years)	15–19	30 (8.7)	4 (1.2)	2 (0.6)	36 (10.5)
	20–34	206 (59.9)	42 (12.2)	24 (7)	272 (79.1)
	35–49	18 (5.2)	12 (3.5)	6 (1.7)	36 (10.4)
Birth order	1	62 (18)	12 (3.5)	4 (1.2)	78 (22.7)
	2–3	122 (35.5)	26 (7.5)	18 (5.2)	166 (48.2)
	4–5	52 (15.1)	16 (4.7)	2 (0.6)	70 (20.4)
	6+	18 (5.2)	4 (1.2)	8 (2.3)	30 (8.7)
Family monthly income ($US)	<25	84 (24.4)	26 (7.6)	18 (5.2)	128 (37.2)
	25–100	146 (42.4)	18 (5.2)	12 (3.5)	176 (51.1)
	>100	24 (7)	14 (4.1)	2 (0.6)	40 (11.7)
Women education	No education	66 (19.2)	12 (3.5)	8 (2.3)	86 (25.0)
	Primary	134 (38.9)	44 (12.8)	16 (4.7)	194 (56.4)
	Secondary	34 (9.9)	2 (.6)	8 (2.3)	44 (12.8)
	College/university	20 (5.8)	0 (00.0)	0 (0.0)	20 (5.8)
Knowledge complications	Yes	188 (54.6)	28 (8.2)	22 (6.4)	238 (69.2)
	No	66 (19.2)	30 (8.7)	10 (2.9)	106 (30.8)

N.B: ^a^skilled assistance may include unskilled, ^b^assisted by HEWs, TBAs and other unskilled Health worker, ^c^had home based self –care, left unassisted at all, home assisted by husband

As summarized in Table 3, of the total 254 respondents who had skilled assistance to pregnancy complications, about 170 (72.3 %) respondents had ANC by a skilled provider in the recent pregnancy. Out of 58 respondents who had unskilled assistance to pregnancy complications, about 52 (89.7 %) respondents had unskilled ANC follow up in the recent pregnancy.

**Table 3:** Percent distribution of skilled and unskilled assistance to pregnancy complications by four choices of ANC provider in the recent pregnancy, Arba Minch Zuria district, south west Ethiopia, 2014

ANC provider	Sought assistance		
	Category	Skilled assistance^a^	Unskilled assistance^b^
		*n* (%)	*n* (%)
Skilled ANC	Yes	166 (65.4 %)	4 (6.9 %)
	No	88 (34.6 %)	54 (93.1 %)
	Total	254 (100 %)	58 (100 %)
Unskilled ANC	Yes	84 (33.1 %)	52 (89.7 %)
	No	170 (66.9 %)	6 (10.3 %)
	Total	254 (100 %)	58 (100 %)

N.B: ^a^skilled assistance = had sought health care assistance by doctor, nurse, midwife, or Health Officer

^b^Unskilled assistance = had sought health care assistance by HEWs, TBAs, or other unqualified provider

### Predictors of skilled assistance seeking behaviour

The multivariable analysis carried out using binary logistic regression indicated that four variables: Family monthly income, having ANC by a skilled provider, age of respondents, and transportation access to the facility were the factors found to be significantly associated with seeking assistance from a skilled provider. Women who had antenatal care by a skilled provider found to be 10.6 times (AOR = 10.6, 95% CI; 3.3, 34.5) more likely to seek assistance from a skilled provider for pregnancy complications as compared to women who did not have antenatal care by a skilled provider. Women with household monthly income of $US25-100/month were about 3.4 times (AOR = 3.4, 95 % CI; 1.04, 11.4) more likely to be assisted by a skilled provider at the time of pregnancy complications than women who had monthly household income below $US25. Women who were in the age 20–34 years were about 3.8 times (AOR = 3.8; 95 % CI, 1.2, 12.3) more likely to be assisted by a skilled provider at the time of pregnancy complications as compared to older women (below 20 years of age) with complications. Similarly, women who had access to transportation to go to the facility were 72.2 times (AOR = 72.2; 95 % CI, 17.2, 303.5) more likely to be assisted by a skilled provider for pregnancy complications as compared to those women of the counterparts (Table 4).

**Table 4:** Adjusted and unadjusted odds ratio of logistic regression model showing effects of predictor variables on the likely hood of skilled assistance for pregnancy complications, Arba Minch Zuria district, south west Ethiopia, 2014

Variables		Assisted by skilled provider							
		Yes	No	COR	95 % CI		AOR	95 % CI	
		*n* = 254	n^b^ = 90		Lower	Upper		Lower	Upper
Age (years)	15–19	30	8	5.8	0.99	34.4	4.2	0.6	28.1
	20–34	206	64	3.6*	1.2	10.8	3.8*	1.2	12.3
	35–49	18	18	1.0+			1.0+		
Family Monthly Income ($US)	<25	84	42	1.0+			1.0+		
	25–100	146	32	5.4*	0.6	16.8	3.4*	1.01	11.4
	>100	24	16	2.0	0.68	5.89	5.7	.59	55.5
Had ANC by Skilled Provider	Yes	166	22	12.7*	4.1	38.7	10.6*	3.3	34.5
	No	88	68	1.0+			1.0+		
Knowledge of Complications	Yes	188	62	0.23^a^	0.1	0.52	1.13	0.2	6.4
	No	66	28	1.0+			1.0+		
No transport access problem	Yes	198	6	33*	9.3	116.6	72.2*	17.2	303.5^c^
	No	56	84	1.0+			1.0+		

N.B: *Statistically Significant Association (*p* < 0.05)

^a^Significant by binary analysis

^b^includes unskilled assistance, or self care or no assistance at all

^c^large upper limit

+ Reference category

## Discussion

This study showed, 43.1 % of the respondents had encountered any one or more of the pregnancy complications. This finding was lower from an Indian slum study (43.1 % VS 60 %) [7], but higher from that of North Ethiopian study which was 28.5 % [9]. This increased perception for reporting may be related to increased health extension workers and the recent network of health development army in the community and the increase in health services infrastructures in Ethiopia. Lower finding comparative to Indians may show low reporting of the complications & its low perceived severity as related to Indian pregnant women’s, though had increasing perception and reporting seen from time to time in Ethiopia.

In this study, skilled ANC attendance and skilled assistance seeking to pregnancy complications was significantly associated (AOR = 10.6, 95 % CI; 3.3, 34.5). This finding was consistent with other studies in that the contribution of ANC by skilled provider to further maternal service utilizations was observed in previous studies in Ethiopia [9, 20] and outside Ethiopia [13, 21]. This may be due to effective counseling services given to pregnant women during ANC visits by a skilled provider better motivated the women for further assistance as compared to unskilled ANC provider (HEW and TBA). There is no doubt that this is because skilled providers were more skillful than unskilled providers for effective and more quality counseling on pregnancy complications and its symptoms.

This study revealed that some of the women who reported complications did not seek services due to reasons, like did not see seriousness of the problems, seen as not necessary, and because family did not allow to seek assistance. These finding agreed with the findings outside Ethiopia [7, 12, 22], but differed with the previous findings of home study [9] and outside [11] in that inability to judge the graveness of condition, distance/transport problems, lack of money/cost considerations, and use of traditional options at home, inadequate training of health personnel and lack of health insurance were observed. These differences may be due to cultural differences of inhabitants from region to region with in the home country and some cultural similarities of home country with outside home to influence on not to seek assistance to pregnancy complications.

In current study, it was identified that there was statistically significant association between medium household monthly income and skilled assistance to pregnancy complications (AOR = 3.4, 95 % CI; 1.04, 11.4). This was in-line with previous findings at home [9], while not in agreement with the findings abroad [6, 7, 15] in that skilled assistance was higher among wealthier households. The difference may be due to difference in income classifications of the studies. Household monthly income of this study was classified as below $US 25, $US 25–100 and above $US 100.

Women in the age group 20–34 years old had statistically significant association with seeking skilled assistance to pregnancy complications (AOR = 3.8; 95 % CI, 1.2, 12.3). This was not agreeing Indian study finding [7] in that more use of skilled assistance observed with greater maternal age, such as more than 35 years. The difference could be due to high number of women in the last one year post birth exist in the age group of 20–34 years in this study district as compared to Indians outside finding. Moreover, the increased primary education in Ethiopia might have contributed for the increased seeking behavior.

Women who had transportation access to the health facility had statistically significant association (AOR = 72.2; 95 % CI, 17.2, 303.5) with skilled assistance by a skilled provider. This is in-line with the findings in Upper(southern) Rural Egypt study [16] in that transportation service accessibility problems were seen as barrier to seeking skilled assistance to pregnancy complications in both studies.

## Conclusion and recommendations

Women who reported at least any one of the problems faced in the recent pregnancy were highest as compared to previous prevalence. The most common complications reported were Malaria, Nausea/Vomiting and severe head ache. Of women faced complications, more than seven out of ten sought assistance from a skilled provider. Belonging to income group $US25 to 100, getting antenatal care from a skilled provider, maternal age of 20–34 years, transport access were independent predictors of seeking assistance from a skilled provider. Community awareness program should focus on life threatening pregnancy complications on seeking assistance.

### Strengths of the study

Being community based is an advantage for representing the community of woreda as compared to being facility based. Moreover, Professional data collectors (being midwifery nurse) used was an advantage for effective collection of medical information from the respondent’s.

### Limitations of the study

The cause effect relationship for all significant associations may have a chicken egg dilemma, since this is cross-sectional study. The reliability of self-reported complications based on a woman’s recall may be limited compared to the results of medical examinations at health care facilities. Judging the severity of the illness is also a challenge to respondents, since severity can be subjective, even though women’s self-report will be the primary option for capturing maternal morbidity in places where women do not usually use skilled care providers or facilities for maternal services. The fact that women who died from obstetric complications were excluded from this study might have some effect on this finding.
